# Learned value modulates the access to visual awareness during continuous flash suppression

**DOI:** 10.1038/s41598-023-28004-5

**Published:** 2023-01-14

**Authors:** Claudia Lunghi, Arezoo Pooresmaeili

**Affiliations:** 1 Laboratoire Des Systèmes Perceptifs, Département d’études Cognitives, École Normale Supérieure, PSL University, CNRS, 29, Rue d’Ulm, 75005 Paris, France; 2 Perception and Cognition Lab, European Neuroscience Institute Goettingen- A Joint Initiative of the University Medical Center Goettingen and the Max-Planck-Society, Grisebachstrasse 5, 37077 Goettingen, Germany

**Keywords:** Cognitive neuroscience, Consciousness, Motivation, Perception

## Abstract

Monetary value enhances visual perception and attention and boosts activity in the primary visual cortex, however, it is still unclear whether monetary value can modulate the conscious access to rewarding stimuli. Here we investigate this issue by employing a breaking continuous flash suppression (b-CFS) paradigm. We measured suppression durations of sinusoidal gratings having orthogonal orientations under CFS in adult volunteers before and after a short session of Pavlovian associative learning in which each orientation was arbitrarily associated either with high or low monetary reward. We found that monetary value accelerated the access to visual awareness during CFS. Specifically, after the associative learning, suppression durations of the visual stimulus associated with high monetary value were shorter compared to the visual stimulus associated with low monetary value. Critically, the effect was replicated in a second experiment using a detection task for b-CFS that was orthogonal to the reward associative learning. These results indicate that monetary reward facilitates the access to awareness of visual stimuli associated with monetary value probably by boosting their representation at the early stages of visual processing in the brain.

## Introduction

In order to interact efficiently with the external environment, our brain needs to select the most relevant information amongst all the available sensory cues that compete for neural resources^1^, gating its access to perceptual awareness. One important factor modulating perceptual selection is motivation, which can be efficiently manipulated via reward^2–5^.

Several studies have shown that monetary reward can modulate sensory processing in different modalities, both at the perceptual and neural level. For example, in the visual modality, monetary value enhances visual sensitivity^3,6–8^, attentional selection^2^, visual perceptual learning^9–11^, binocular rivalry^12,13^ and modulates activity in early visual areas^6,14–17^. However, it is unknown whether and how reward modulates the conscious access of visual information associated with monetary value. To answer this question, we tested whether learning an arbitrary association between visual features and monetary reward can alter the suppression duration of visual stimuli rendered invisible by interocular suppression in a breaking Continuous Flash Suppression (b-CFS) paradigm^18,19^. CFS is a psychophysical technique that allows the prolonged perceptual suppression of low-contrast visual targets presented to one eye by the simultaneous presentation of high-contrast dynamic masks to the other eye^20^.

One advantage of using CFS compared to other forms of visual bistability (such as binocular rivalry^21–23^) is that CFS specifically measures the time taken by visual information to access visual awareness. In binocular rivalry, in contrast, one cannot disentangle the faster access to awareness of stimuli presented to one eye, from the maintenance in awareness of the stimuli presented to the other eye. For example, in binocular rivalry increasing the stimulus strength in one eye increases the overall predominance of that eye and shortens the predominance of the other eye^24,25^. In this scenario, it is difficult to untangle access to awareness and maintenance in awareness, and therefore to interpret the mechanism underlying the increased predominance of rewarded visual stimuli previously shown in binocular rivalry^12,26^.

The suppression of a visual target during CFS reflects the time necessary for the visual stimulus to reach awareness and depends on the strength of its signal (but see^18,19,27–29^ for post-perceptual effects). For example, visual stimuli having high contrast show shorter suppression durations compared with low-contrast visual stimuli^20,30,31^. As monetary reward has been shown to enhance visual sensitivity of stimuli associated with high rewards^3,6–8^, we hypothesized shorter suppression durations for the visual stimulus associated with high monetary value in a b-CFS paradigm after learning the association between a particular visual feature and monetary value. This would indicate that monetary value associated with a suppressed visual stimulus accelerates its emergence to visual awareness probably by boosting its signal strength.

## Materials and methods

### Subjects

Twenty subjects (10 females, average age ± standard deviation: 25 ± 5 years), participated in experiment 1. Twenty-eight subjects (9 males, average age ± standard deviation: 30 ± 7 years), participated in experiment 2, two participants were excluded because of poor accuracy (< 75%) in the b-CFS task. A total of 26 participants were therefore included in the analyses of the data of experiment 2. All subjects had normal or corrected-to-normal visual acuity (ETDRS charts) normal stereoacuity (TNO) and normal color vision (Ishihara plates). All subjects were naïve to the purposes of the experiment. Sample size was determined based on a previous experiment^6^, in which the same Pavlovian associative learning protocol was used to reveal an effect of monetary reward on visual perception. Because of time constraints, sample size was lower for experiment 1 than for experiment 2.

### Ethic statement

The experimental protocol was approved by the local ethics committee (Comité d’éthique de la Recherche de l’université Paris Descartes, CER-PD:2018–55-LUNGHI) and was performed in accordance with the Declaration of Helsinki (DoH-Oct2008). All participants gave written informed consent.

### Apparatus and stimuli

The experiments took place in a quiet and dark room. The visual stimuli were developed in MATLAB (The MathWorks Inc., Natick, MA) using Psychtoolbox-3 ^32^ running on a PC (Alienware Aurora R8) and a NVIDIA graphics card (GeForce RTX2080). Visual stimuli were presented dichoptically through a custom-built mirror stereoscope and subjects’ head was stabilized with a forehead and chin rest.

#### Continuous flash suppression

**Figure 1:**
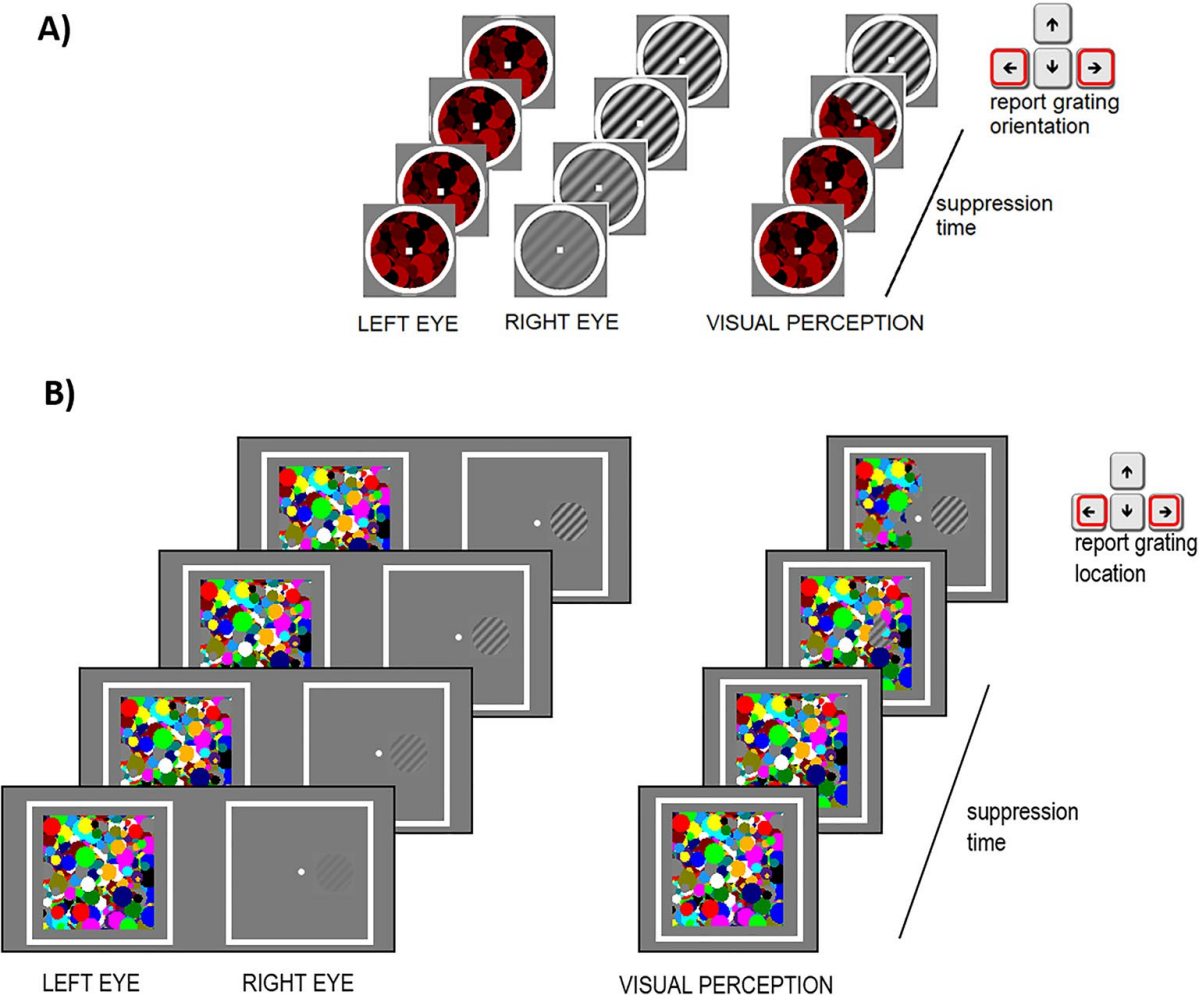
Breaking continuous flash suppression (b-CFS) experimental paradigm. (**A**) breaking Continuous Flash Suppression (b-CFS) task in Experiment 1. Observers reported the orientation of the grating using the appropriate keyboard keys as soon as it emerged from interocular suppression induced by a dynamic Mondrian patterns flashed in the other eye at 10 Hz. (**B**) b-CFS task in Experiment 2. Observers reported the location of the grating as soon as it emerged from interocular suppression.

Experiment 1. The stimuli consisted of red dynamic masks^33^ and luminance-modulated target gratings. The stimuli were presented on a uniform grey background (luminance: 17.1 cd/m^2^, CIE *x* = 0.294, *y* = 0.316) in central vision, surrounded by a white smoothed circle (See Fig. 1A). The target stimulus was one of two orthogonal sinusoidal gratings (orientation: ± 45°, diameter: 2.5°, spatial frequency 2 cycles/degree). A set of 50 independent Mondrian-like masks were pre-generated and served as suppressors. These were presented to one eye at a rate of 10 Hz, meaning that the suppressor was refreshed with an independently drawn suppressor from the set of Mondrians every 100 ms. Mondrian masks were generated by randomly positioning 64 minimum, maximum, and medium luminance red circles of different size (ranging from 0.12° to 0.54°). For an example of the visual stimuli used in the experiment, see Fig. 1A. The visual stimuli in each eye contained a small (0.2°) white fixation square in their center and were presented on a linearized 24-inch LCD display (BenQ XL2420z), driven at a resolution of 1920 × 1080 pixels and at a frame rate of 60 Hz. To facilitate binocular fusion, the visual stimuli were embedded into a white squared frame (size 4 degrees of visual angle).

Experiment 2. The visual stimuli and the experimental setup were identical to experiment 1 except that the mask stimuli subtended 6 degrees of visual angle and consisted of circles having different colors. The target stimuli were presented 1.75° from fixation, either on the left or right visual field. For an example of the visual stimuli used in the experiment, see Fig. 1B. The visual stimuli in each eye contained a small (0.2°) white fixation square in their center. To facilitate binocular fusion, the visual stimuli were embedded into a white squared frame (size 9 degrees of visual angle).

### Experimental procedure

**Figure 2:**
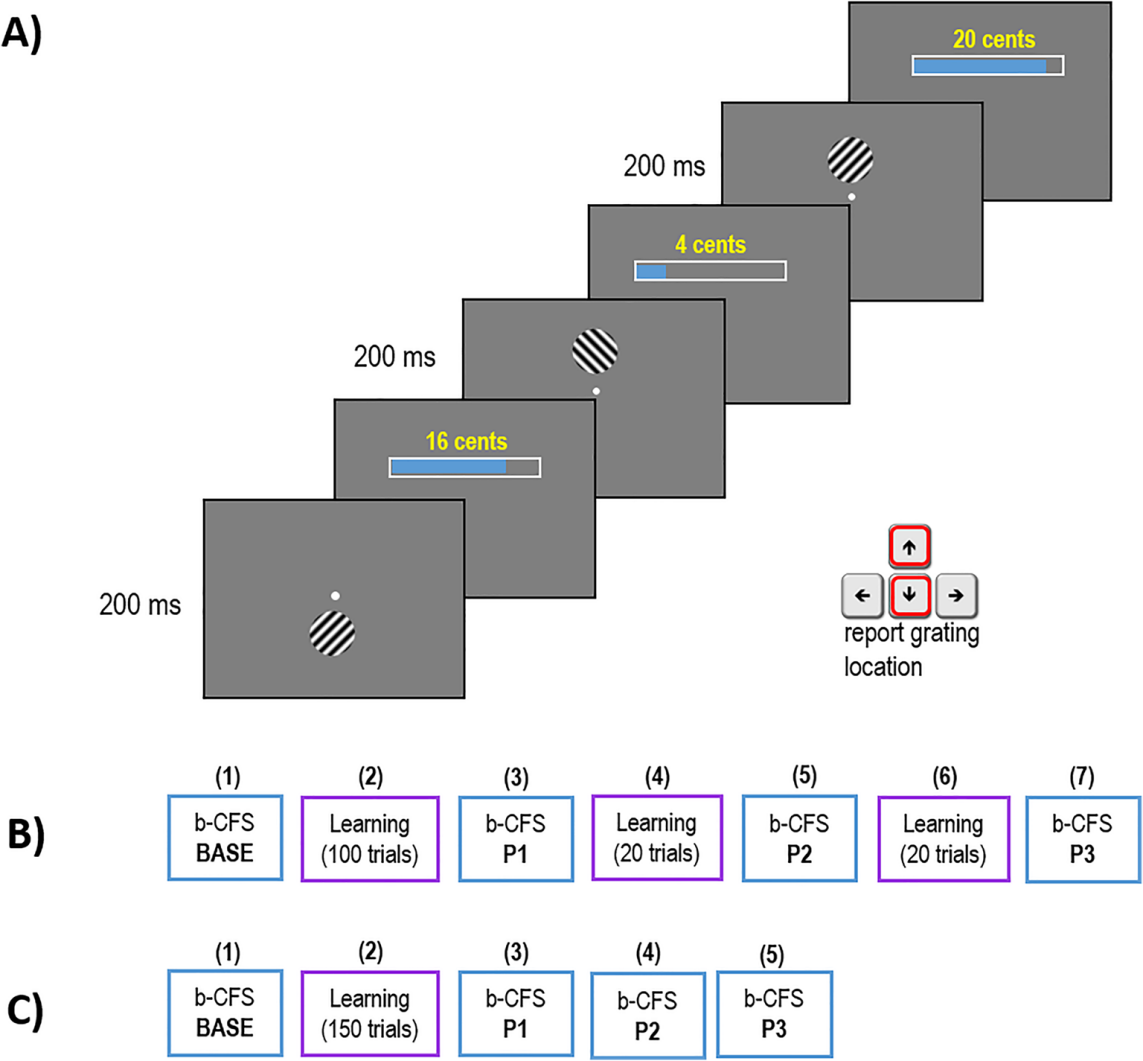
Pavlovian associative learning paradigm and experimental pipeline. (**A**) Associative learning task. Observers learned the association between each of two possible visual orientations and either high or low monetary reward using a Pavlovian conditioning paradigm. (**B**) Experimental pipeline for Experiment 1. (**C**) Experimental pipeline for Experiment 2. BASE = baseline session, P1-P2-P3 = post-learning sessions.

Experiment 1 consisted of seven different blocks performed consecutively, the two tasks (breaking continuous flash suppression, b-CFS, and reward learning) were alternated. The first block (b-CFS base) consisted of 30 trials and was followed by 100 trials of reward learning. After the reward learning task, three different 30-trials bCFS blocks were performed (P1-P2-P3) alternated by a short (20 trials) recall block of reward learning (Fig. 2B). Before starting the experiment, observers were familiarized with the two tasks (b-CFS and Reward learning) with a short training (10 trials per task). Experiment 2 consisted of five different blocks performed consecutively. The first block (b-CFS base) consisted of 40 trials and was followed by 150 trials of reward learning. After the reward learning task, three different 40-trials bCFS blocks were performed (P1-P2-P3, Fig. 2C). Before starting the experiment, observers were familiarized with the two tasks (b-CFS and Reward learning) with a short training (10 trials per task).

#### Continuous flash suppression

Experiment 1. Each experimental block consisted of 30 trials. Before each block only the white squared frames were presented, allowing the subject to align the mirrors of the stereoscope to achieve perfect fusion. Subjects self-initiated each trial by button press. The dichoptic CFS stimuli (a grating target to one eye and a dynamic Mondrian mask to the other) appeared 500 ms after the button press. In order to ensure invisibility of the target grating, its contrast was ramped from 0 to the condition’s maximum contrast (23% Michelson contrast) over a period of 7.5 s following an on-ramp with a half-Gaussian profile (sigma = 0.4, beta = 0.05). A rather long contrast ramp and a relatively low maximum contrast were chosen to maximize the invisibility of the target in the CFS paradigm. The observer’s task was to report the orientation of the visual grating (left or right, pressing the corresponding keyboard button) as soon as it broke through CFS suppression and became visible. The observer’s button-press determined the end of the trial. The orientation of the target grating and the eye of presentation were randomized on each trial.

Experiment 2. Each experimental block consisted of 40 trials. Before each block only the white squared frames were presented, allowing the subject to align the mirrors of the stereoscope to achieve perfect fusion. Subjects self-initiated each trial by button press. The dichoptic CFS stimuli (a grating target to one eye and a dynamic Mondrian mask to the other) appeared 500 ms after the button press. In order to ensure invisibility of the target grating, its contrast was ramped from 0 to the condition’s maximum contrast (23% Michelson contrast) over a period of 7.5 s following an on-ramp with a half-Gaussian profile (same as experiment 1). The observer’s task was to report the location of the visual grating (left or right from fixation, pressing the corresponding keyboard button) as soon as it broke through CFS suppression and became visible. The observer’s button-press determined the end of the trial. The orientation of the target grating and the eye of presentation was randomized on each trial.

#### Associative reward learning

##### Main condition

We used a Pavlovian conditioning paradigm as shown in Fig. 2A to familiarize subjects with grating orientations and their associated rewards. For each trial, a sinusoidal grating (size 2.5°, spatial frequency 2 cpd, contrast 23% Michelson contrast) was briefly (250 ms) presented 4° from the fixation spot either on the upper or lower visual field on a grey background (luminance: 17.1 cd/m^2^, CIE *x* = 0.294, *y* = 0.316). Subjects indicated the location of the grating relative to the fixation point (upper or lower) with a key press. Contingent on correct performance, subjects received a reward shown both graphically and with Euro coin numbers (e.g., 10 cents). We used two orientations (± 45°) corresponding to the grating orientations in the b-CFS task, each paired with high or low monetary reward levels, so that either the left-tilted or right-tilted orientation was consistently rewarded with higher values and vice versa. Reward values had a Poisson distribution, with two different λ values (mean and variance are equal in a Poisson distribution), 3 cents for low rewards and 30 cents for high rewards. The pairing of reward (high or low) with the grating orientation was counterbalanced across subjects. Subjects performed this “grating localization” task and learned the orientation–reward pairing, as verified in debriefings after the experiment. In the debriefing, two visual stimuli were presented consecutively and participants were required to indicate which one of the two had been associated with high monetary reward during the learning section. Across the two experiments, 32 out of 40 participants correctly identified the association between monetary reward and orientation created during the Pavlovian associative learning.

### Analyses

Suppression times for the target gratings were computed for each experimental block as the mean time elapsed between the visual stimulus presentation and the reported visibility of the grating. Because the suppression duration distribution is skewed to the right, suppression durations were log-transformed prior to analyses. The choice of data trimming in b-CFS paradigm has a dramatic impact on the results^34^, for this reason, we adopted a rather conservative approach when removing outlier durations from the analyses. Outliers were defined as suppression durations shorter than 0.5 s or greater than ± 4 SD from the average duration for each subject (the average suppression duration was computed per each subject across all experimental blocks, 0.5% of trials for experiment 1 and 0.6% of trials for experiment 2, were excluded on average).

To assess the effect of associative learning on visual awareness, suppression durations measured after the associative reward learning block were compared to durations measured before learning (baseline) for each orientation using paired-samples t-tests. The Bonferroni-Holm correction for multiple comparisons was applied. Moreover, to directly compare the effect of the association with either high or low monetary reward, for each participant, we normalized suppression durations measured after learning (P1-P2-P3) to those obtained before learning (BASE). We then performed a repeated-measures ANOVA with the factors Reward (High or Low) and Experimental block (P1-P2-P3). Should the association with either high or low monetary reward affect suppression duration under CFS, we expect to observe a main effect of the factor reward.

## Results

We tested the effect of monetary reward on visual awareness in two different experiments by measuring the suppression times of orthogonally oriented gratings in a breaking Continuous Flash Suppression (b-CFS, Fig. 1) paradigm. B-CFS was tested before and after a learning session during which monetary reward (high or low) was associated to either orientation (Fig. 2).

### Experiment 1

**Figure 3:**
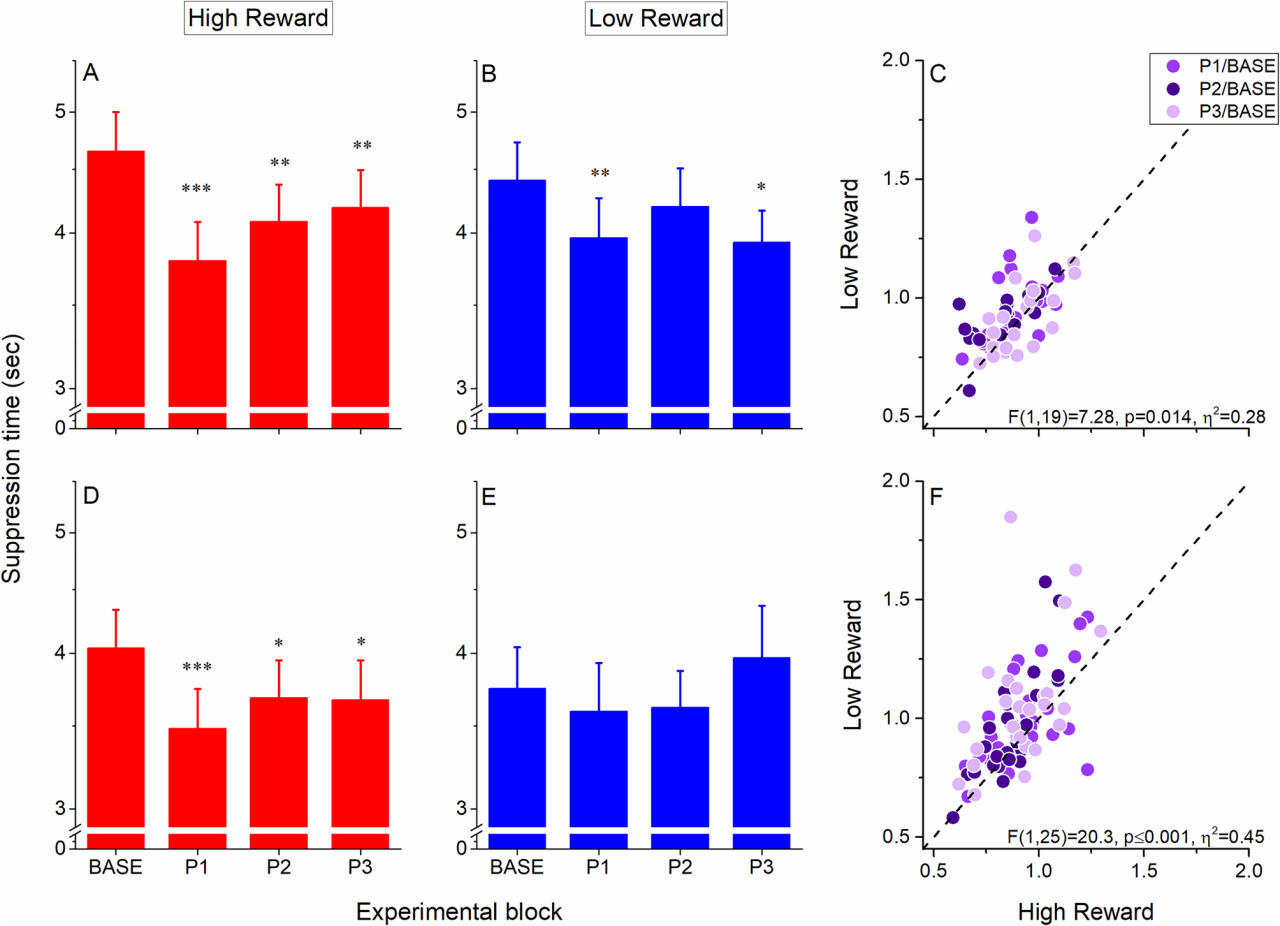
Effect of monetary reward on conscious access. Data from experiment 1 showing suppression times acquired in the b-CFS paradigm before (BASE) and after (P1-P2-P3) associative learning between visual stimuli orientation and monetary reward. Average (*N* = 20) suppression times of visual stimuli associated with high (**A**) and low (**B**) monetary reward in Experiment 1. Error bars represent 1 ± S.E.M. Suppression times were analyzed after a log10 transformation, for this reason, the y axis is in log10 scale. (**C**) scatter plot of the ratio between suppression times measured after (P1, P2 and P3, symbols with progressive shades of purple) and before (BASE) associative reward learning for stimuli associated with High vs Low monetary reward in Experiment 1. (**D-F**) Same as A-C for Experiment 2 (*N* = 26). Asterisks represent the significance level (Bonferroni-Holm correction for multiple comparisons applied) of post-hoc tests for P1, P2 and P3 vs BASE: *** = corrected *p* < 0.001, ** = corrected *p* < 0.01, * = corrected *p* < 0.05.

We found that after a brief learning session (100 trials) in which participants associated high or low monetary reward to each of two orthogonal orientations, the suppression time of the orientation arbitrarily associated with either high (Fig. 3A) or low (Fig. 3B) monetary reward was shorter compared to baseline measurements, with a larger effect being observed for high monetary reward. For the visual stimulus associated with high monetary reward, the effect of associative learning was significant for all three sessions after learning (Fig. 3A): in the first session after learning (P1), the suppression time of stimuli associated with high reward was on average 868 ± 170 ms shorter compared to baseline (t(19) = 5.68, corrected *p* < 0.0001), in the second session (P2) suppression times decreased on average of 568 ± 155 ms (t(19) = 3.79, corrected *p* = 0.002), and of 460 ± 161 ms in the third session (P3, t(19) = 3.08, corrected *p* = 0.006). Suppression times associated with low monetary reward (Fig. 3B) were significantly lower for the P1 (444 ± 124 ms, t(19) = 3.71, *p* = 0.004) and P3 (474 ± 172 ms, t(19) = 2.78, *p* = 0.024), but not for the P2 (209 ± 169 ms, t(19) = 1.29, *p* = 0.21) experimental block after associative learning, confirming that the effect of associative learning was overall lower for visual stimuli associated with low monetary reward compared with high monetary reward. A repeated-measures ANOVA on the suppression times obtained after learning normalized to the baseline suppression times revealed a significant main effect of the factors REWARD (F(1,19) = 7.28, *p* = 0.014, is η^2^ = 0.28) and Experimental Block (F(2,19) = 4.22, *p* = 0.022, is η^2^ = 0.18), as well as a significant interaction between the two factors (F(2,38) = 3.35, *p* = 0.046, is η^2^ = 0.15), indicating a specific effect of the amount of monetary reward value associated to the visual stimuli during learning and a decay of the effect over time, despite the short blocks of recall learning. Figure 3C shows individual subjects’ data normalized to the baseline suppression times for visual stimuli associated with high and low monetary reward.

### Experiment 2

In experiment 1, observers were asked to report the visibility of the grating in the b-CFS paradigm indicating its orientation (clockwise or counter-clockwise), see Fig. 1A. Since the response collected in the b-CFS paradigm was not orthogonal to the associative learning, we conducted a second experiment in which, in the b-CFS paradigm, we asked observers to report the location of the target grating (right or left to fixation, see Fig. 1B). This detection task was orthogonal to the visual feature associated with monetary reward, decreasing the possibility of an involvement of post-perceptual factors and response bias in driving participants’ performance. Moreover, since the effect of associative learning was short-lived, in experiment 2 we also removed the recall learning session in between the b-CFS blocks recorded after the main learning condition.

In experiment 2 we observed a slightly different pattern of results compared to experiment 1: after associative learning, the suppression duration of visual stimuli associated with high (Fig. 3D) monetary reward decreased compared to baseline measurements, while suppression times of the stimulus associated with low reward (Fig. 3E) remained unchanged. For the visual stimulus associated with high monetary reward, the effect of associative learning was significant for all three sessions after learning (Fig. 3D): in the first session after learning (P1), the suppression time of stimuli associated with high reward was on average 559 ± 115 ms shorter compared to baseline (t(25) = 5.1, *p* < 0.0001), in the second session (P2) suppression times decreased on average of 353 ± 163 ms (t(25) = 2.37, *p* = 0.026), and of 368 ± 144 ms in the third session (P3, t(25) = 2.73, *p* = 0.023). No significant difference was observed between baseline and post-training suppression times for the stimulus associated with low monetary reward (Fig. 3E, all ps > 0.34). The lack of an effect for the low-reward condition observed here might be a result of the fact that, in Experiment 2 a CFS task orthogonal to the reward manipulation was used, reducing the reinforcing effect of a similar task during the reward training and the main task and hence the potential response bias effects. Consistently with Experiment 1, a repeated-measures ANOVA on the suppression times measured after learning and normalized to the baseline revealed a significant main effect of both the factors Reward (F(1,25) = 20.33, *p* < 0.001, is η^2^ = 0.45) and Experimental block (F(2,25) = 4.3, *p* = 0.019, is η^2^ = 0.15), but no significant interaction between the two (F(2,50) = 1.15, *p* = 0.32, is η^2^ = 0.04). This result indicates that in Experiment 2 the specific effect of monetary reward value on associative learning was confirmed, but that this effect did not decay over time, despite the fact that no recall learning sessions were performed between the experimental blocks. Figure 3F shows individual subjects’ data normalized to the baseline suppression times for visual stimuli associated with high and low monetary reward.

## Discussion

We found that learned value modulates visual awareness by promoting the release of visual stimuli associated with monetary reward from interocular suppression in a breaking Continuous Flash Suppression (b-CFS) paradigm. Visual stimuli associated with high monetary reward emerged earlier to visual awareness compared to visual stimuli associated with low monetary reward. This effect was replicated in a separate experiment performed in an independent sample of participants, using the same analysis pipeline, but a detection task in the CFS experiment that was orthogonal to the visual feature associated with monetary reward.

Previous work has shown that reward value influences visual processing both at the perceptual and at the neural level^3^. For example, reward enhances visual sensitivity^6^ and visual adaptation^35^, modulates probability information processing^36^, time perception^4^, and binocular rivalry^12,13,26^.Reward also alters ocular movements^37–39^ and improves perceptual learning^9–11^. At the neural level, it has been shown that reward signals can modulate activity in the early visual cortex^6,14–17,40,41^. Here we provide the first evidence that information about object value can modulate the conscious access of rewarding visual stimuli in a b-CFS paradigm. In particular, we show a specific effect of the amount of monetary value associated with a visual stimulus on suppression times during CFS: suppression times were consistently reduced for stimuli associated with high monetary reward, compared to stimuli associated with low monetary reward.

Two previous experiments have indicated that monetary reward can influence visual competition during binocular rivalry, showing that monetary reward delivered during rivalrous perception prolongs the perceptual dominance of the rewarded visual stimuli^12,26^. In our experiment we extended these observation targeting the access to visual awareness. To this aim we used Continuous Flash Suppression^20^, a variant of binocular rivalry, which specifically targets the access of visual information to awareness. Furthermore, these previous studies investigated the effect of monetary reward delivered during binocular rivalry modulating observers’ motivation: larger monetary rewards were obtained when perceiving one of the rivalrous stimuli, motivating the observer to maintain the rewarded stimulus into visual awareness. In our study, instead, monetary reward was obtained only during the associative learning phase, and not during CFS (for a similar Pavlovian paradigm involving threat see^42^). The privileged access to visual awareness of rewarded visual stimuli that we observed therefore reflects the processing of previously associated monetary value rather than an enhancement of motivation to perceive visual stimuli that predict the reward delivery.

Visual suppression during CFS can occur early in the visual system, already at the level of the primary visual cortex^43^. It has been proposed that CFS acts in the early visual cortex by fractionating the representation of suppressed visual stimuli into their basic characteristics^28^. In this vein, the claim that semantic information might be preserved under CFS has been criticized, as the integrated representation of the suppressed visual stimuli is disrupted by CFS^28^. In our experiment, monetary reward was associated with a basic visual feature such as orientation, which is processed at the earliest stages of visual analysis^44^. The fractionation induced by CFS might therefore preserve orientation information, which might be processed during suppression. On the other hand, whether b-CFS paradigms reflect unconscious visual processing is a highly debated issue^18,28,29^: response time difference in b-CFS paradigms might in fact reflect post-perceptual processing modulating response speed rather than visual stimuli detection times. Whereas we cannot completely rule out this possibility, we took care of reducing post-perceptual effects in two ways: first by using relatively low-contrast target stimuli (that are more deeply suppressed) and second by replicating the results in a second experiment, in which we adopted a detection task in b-CFS orthogonal to the reward manipulation. In this vein, the effect of monetary value on the access to visual awareness that we observed likely reflects a perceptual effect possibly occurring early in the visual system, probably through feedback from higher order areas, as previously observed in other experimental paradigms^6,14–17,40,41^.

Finally, monetary reward has been shown to modulate both selective and exogenous attention^2,7,45,46^, making it often difficult to dissociate the two processes^47^. In our experiment, during CFS the observer has no conscious access to the suppressed visual target and cannot voluntarily attend to a particular feature of the stimulus (e.g. its orientation). Furthermore, in a second experiment, we replicated our results when even during the associative learning phase the rewarded features (i.e., location) were not the task-relevant target and therefore were not attended. This is different from other forms of bistability. For example, during binocular rivalry^21–23^ visual perception oscillates between the two monocular images, so that even though at each time one of the two visual stimuli is perceptually suppressed, the observer has some memory about the features of the suppressed visual stimulus from the perceptual history. This knowledge of the suppressed stimulus’ properties could be used to orient selective attention towards the visual stimulus providing monetary reward^12,13^, which was not the case in our experiments, excluding the possibility that the observed effect is entirely mediated by voluntary attention.

Taken together, our results show that learned value of a visual object can modulate visual awareness by boosting the representation of visual stimuli associated with high monetary reward compared to low monetary reward, indicating that value information can be represented before visual awareness and possibly at early stages of visual processing.

## Data Availability

The datasets generated and/or analysed during the current study are available in the Zenodo repository: https://doi.org/10.5281/zenodo.7143926.
